# Covalently Functionalized Cellulose Nanoparticles for Simultaneous Enrichment of Pb(II), Cd(II) and Cu(II) Ions

**DOI:** 10.3390/polym15030532

**Published:** 2023-01-19

**Authors:** Huda Alsaeedi, Hilal Ahmad, Malak Faisal Altowairqi, Afnan Abdullah Alhamed, Ali Alsalme

**Affiliations:** 1 Department of Chemistry, College of Science, King Saud University, Riyadh 11451, Saudi Arabia; 2 Division of Computational Physics, Institute for Computational Science, Ton Duc Thang University, Ho Chi Minh City 700000, Vietnam

**Keywords:** cellulose nanofibers, extraction, heavy metals, pollution, ICP-OES

## Abstract

Cellulose nanoparticles are sustainable natural polymers with excellent application in environmental remediation technology. In this work, we synthesized cellulose nanoparticles and covalently functionalized them with a multi-functional group possessing ligands. The hybrid material shows excellent adsorption properties for the simultaneous extraction of multiple metal ions in the sample preparation technique. The sorbent shows excellent sorption capacity in the range of 1.8–2.2 mmol/g of material. The developed method was successfully employed for the simultaneous extraction of Pb(II), Cd(II) and Cu(II) from real-world samples (industrial effluent, river water, tap and groundwater) and subsequently determined by inductively coupled plasma optical emission spectroscopy (ICP-OES). The method shows a preconcentration limit of 0.7 ppb attributes to analyze the trace concentration of studied metal ions. The detection limit obtained for Pb(II), Cd(II) and Cu(II) is found to be 0.4 ppb.

## 1. Introduction

Heavy metal contamination of water streams is one of the most hazardous pollutions [1,2,3]. Heavy metal ions are extremely mobile, bioaccumulative and biomagnified, which poses a major risk to both the environment and human health [4,5,6,7]. Metals are released directly into the water or indirectly by soil and are frequently invisible [8,9]. Although some metals are necessary as nutrients, many of them have some degree of toxicity and have an adverse effect on the ecosystem [10,11,12]. Several natural phenomena and mostly anthropogenic actions, such as ore mining and processing, fossil fuel combustion and chemical industries, are the major sources of their exposure to humans [13,14,15,16]. Metals are concentrated and redistributed due to human activity in regions that are not naturally metal-enriched [7,17]. Most chemical industries use transition metal catalysts, which are among the cause of their toxicity. The majority of them are primarily present in an inorganic form and were released into the aquatic environment as a result of natural or anthropogenic processes [18,19]. It is difficult to directly analyze metal ions in aqueous solutions using different atomic spectroscopy techniques since the first and second group elements are present, and their trace level concentration introduces spectrum interferences. Implementing sample preparation techniques to remove co-existing ions while also concentrating the analyte ions by lowering sample volume is highly valuable [20].

Solid phase extraction (SPE) is a promising and much sought-after extraction technique. High enrichment factors, good reproducibility, cheap cost, no use of organic solvents and the ability to combine SPE with spectroscopic methods, including online systems, are all features of the approach [21,22]. With the use of a suitable solid adsorbent, SPE also permits obtaining a multi-element determination of analytes. In the context of trace analysis, novel adsorbents with numerous functional groups for metal ion separation could be useful for this purpose. Activated carbon, poly vinyl chloride-based membranes functionalized with dithizone, ionic liquid immobilized on multi-walled carbon nanotubes, ion imprinted sponge and graphene oxide, and cross-linked sulfhydryl-functionalized graphene oxide are just a few examples of the conventional and novel adsorbents that have recently been published in papers [23,24,25,26,27]. However, a lot of the recently created compounds are either restricted to a few specific metal ions or require greater analyte extraction concentrations. This restricts their use in single-element analysis. Additionally, many environmental samples, such as river flows and industrial wastewaters, might include several metal ions. As a result, it is preferable to carry out heavy metal ion extraction simultaneously, which might greatly simplify and cut down on sample preparation time. 

Numerous bio-sorbents have been examined for their ability to remove metal ions, including cellulose, chitosan, rice husk and modified leaves [28,29,30,31]. However, they displayed decreased adsorption capacity because there were fewer chelating groups available for complexation with heavy metal ions. Therefore, to improve the adsorption performance, their morphological and chemical alterations are needed. *Acetobacter xylinum* bacteria produce cellulose nanoparticles (CNP), which are extracellular cellulose. CNP shares a fundamental structure with plant cellulose that is very hydrophilic and biodegradable [32,33]. CNP is made up of hemicellulose- and lignin-free ultrafine microfibrils with a ribbon-like shape. The ligand immobilization increases CNP’s efficiency in separation, which will be linked to hydroxyl groups on its backbone. In this study, we chemically functionalize the CNP with ethylenediaminetetraacetic acid-linked polyethyleneimine (EDTAPEI) ligand. The adsorption characteristics of CNP are improved by the addition of organic ligands, and chelation, as opposed to physical contact, is typically used to adsorb multi-metal ions. The newly immobilized CNP, which has a higher adsorption capacity than undeveloped CNP, forms chelates with Pb(II), Cd(II) and Cu(II). Due to added surface groups, this novel material is very hydrophilic and exhibits quicker extraction of metal ions from aqueous solutions. With good reproducibility in the adsorption properties, the suggested approach significantly preconcentrates trace metal ions from dilute samples. The emulsion formation, use of carcinogenic organic solvent, phase separation, analyte loss during multi-step extraction, and other problems are all eliminated. By increasing the hydrophilicity and accessibility of metal ions for quicker and simultaneous complexation, the surface groups added to CNP played a crucial part.

## 2. Experimental Section 

### 2.1. Materials and Chemicals

The chemicals used are of analytical reagent (AR) grade and were obtained from commercial chemical suppliers: Cellulose powder, ethylenediaaminetetracetic acid, polyethyleneimine, acetic acid (≥99.7%), sodium hypophosite (≥98%), epichlorohydrin and ethanol (≥99.5%) (Sigma-Aldrich, Burlington, MA, USA). Solutions for pH adjustments were prepared using nitric acid and sodium hydroxide (Sigma-Aldrich and Merck, Burlington, MA, USA), hydrochloric acid (Acros Organics, Fair Lawn, NJ, USA), and standard solution (1000 mg L^−1^) of all metal ions are prepared from their respective salts (nitrate and acetate) in deionized water. Working solutions of desired concentrations are prepared from standards after successive dilutions. All glassware was cleaned using 2% nitric acid before use.

### 2.2. Synthesis of Nanosorbent

#### 2.2.1. Synthesis of EDTAPEI 

First, 10.0 mL of alkali solution and 5.0 g of EDTA and 3.80 g of polyethyleneimine were dissolved. Next, the reactants were mixed with 1.00 g of sodium hypophosphite (SHP). The mixture was then agitated for 4.0 h at 105.0 °C. The mixture was cooled to room temperature following the reaction, and the pale-yellow liquid was identified as an EDTAPEI chelating ligand.

#### 2.2.2. Synthesis of CNP/EDTAPEI 

**Figure 1:**
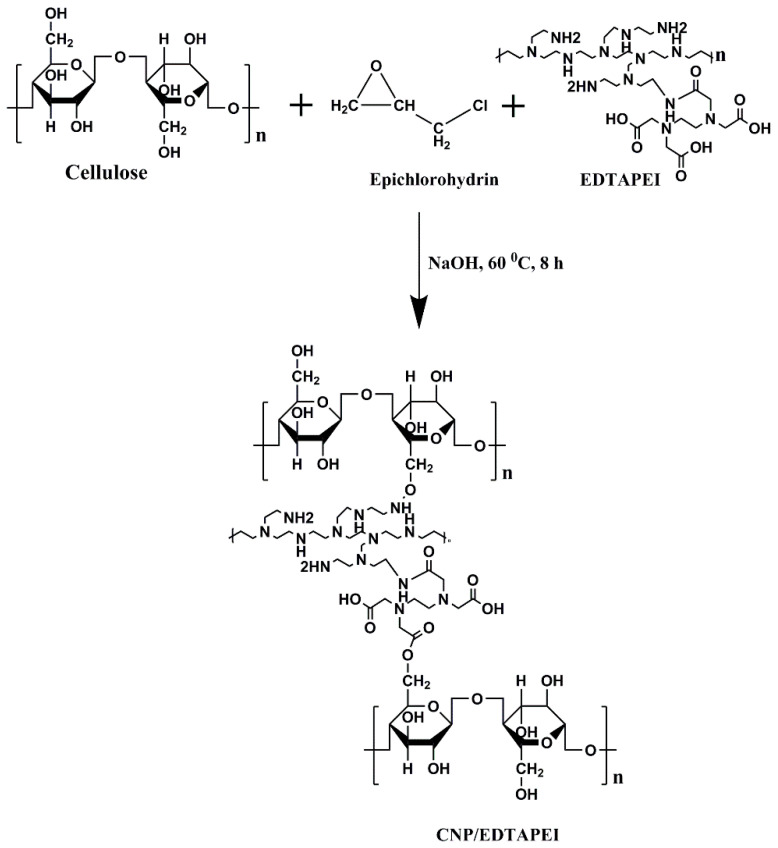
Schematic representation of CNP/EDTAPEI.

To 5.0 mL of alkali solution, 5.0 g of cellulose powder and 10.0 mL of EDTAPEI were first dissolved, and 24.0 mg of epichlorohydrin (ECH) was then added dropwise. The entire reaction mixture spent 8 h in a hydrothermal assembly at 60.0 °C. EDTAPEI and nascent cellulose were cross-linked to create solid particles. Once the pH of the filtered liquid was about neutral, the particles were rinsed with flowing distilled water. After drying the solid components at 60.0 °C for 12 h, the adsorbent was marked as CNP/EDTAPEI. Figure 1 represents the schematic diagram of CNP/EDTAPEI synthesis.

### 2.3. Characterization 

Attenuated total reflection Fourier transform infrared spectra (ATR-FTIR) was recorded for both the ligand and sorbent using (Shimadzu, Torrance, CA, USA). The CNP/EDTAPEI was characterized by high-resolution transmission electron microscopy (HRTEM, Techno, FEI, Tokyo, Japan). The sample was prepared by dispersing sorbent in ethanol before casting on the TEM grid. A Micromeritics Gemini 2380 analyzer (Microtrac, Burladingen, Germany) was used for surface area measurements. Metal ions concentrations were determined using an inductively coupled plasma optical emission spectrometer (ICP-OES, Perkin Elmer Avio 200, Waltham, MA, USA).

### 2.4. Sample Preparation

Industrial effluent, groundwater, tap water and river water samples were collected from the local industrial area. The water samples after the collection were filtered off using a cellulose nitrate membrane (Pore size: 0.22 µm) to remove solid particles and stored in polyethylene bottles before analysis.

### 2.5. Batch Mode of Operation

The effects of sample pH, maximum adsorption capacity and the equilibrium time to reach saturation were studied by taking individual metal ions in batch mode. The rest of the adsorption experiments were conducted in columns. In batch mode, 100 mg of the functionalized CNP/EDTAPEI sorbent was equilibrated with 50 mL of metal ion solution (500 mg L^−1^) maintained at the suitable pH. The solution mixture was stirred at 27 °C for 12 h. After the sorption, the sorbent was filtered off, and the concentration of metal ions in the solution after the dilution was analyzed by ICP-OES.

The equilibrium metal ions adsorption capacity of functionalized CNP/EDTAPEI was calculated from the following equation.

$$Q_{e=}\frac{(C_{o-}C_{e})\;V}{m}$$

where *C*_o_ and *C_e_* (mg L^−1^) are the initial and equilibrium metal ion concentrations, respectively. *V* is the total sample volume (50 mL), and m is the amount of adsorbent dosage (100 mg). 

### 2.6. Column Procedure for Simultaneous Sorption of Metal Ions 

In a column technique, 100 mg of functionalized CNP/EDTAPEI sorbents were packed into a glass column with an inner porous disc that had an 8 mm diameter and a 10 cm length. All column adsorption tests use such columns, which have a bed height of 1.2 cm above the inner porous disc and were preconditioned with 5 mL of a pH 6 ± 0.2 buffer solution. At a flow rate of 8 mL min^−1^, each 100 mL of model solutions containing 10 mg L^−1^ of the examined metal ion (concurrently and at concentrations beyond World Health Organization and Environmental Protection Agency acceptable limits) were run through the column. A peristaltic pump was utilized to maintain the sample flow. The sorbed metal ion was then eluted with 5 mL of a stripping agent after the column had been washed with deionized water following the sorption process. ICP-OES was used to determine how much of the recovered metal ions were present in the eluent. The power of the ICP-OES is 1.5 kW, the Alumina injector is 2.0, the plasma gas is argon, the flow rate of the plasma gas is 8 L min^−1^, the flow rate of the nebulizer gas is 0.7 L min^−1^, the flow rate of the auxiliary gas is 0.2 L min^−1^, the pressure is 3.2 bar and the read time is 2 mL min^−1^. All samples and blanks were examined during the analyses in triplicate. The concentrations of Pb, Cd and Cu divalent ions’ respective to emission intensities were examined at wavelengths of 220.35, 228.80 and 324.75 nm, respectively.

## 3. Results and Discussion

### 3.1. Characterization

**Figure 2:**
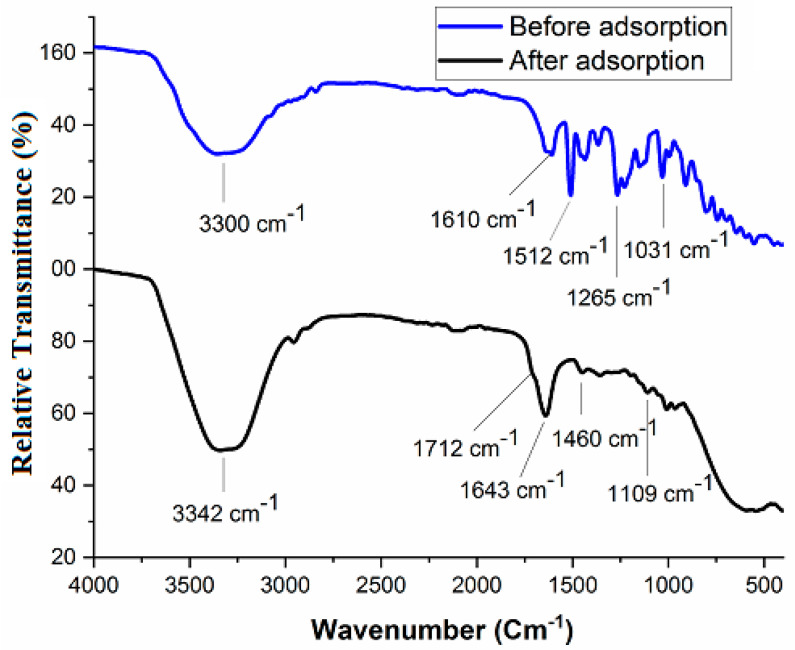
ATR−FTIR of CNP/EDTAPEI sorbent before and after metal ion adsorption.

**Figure 3:**
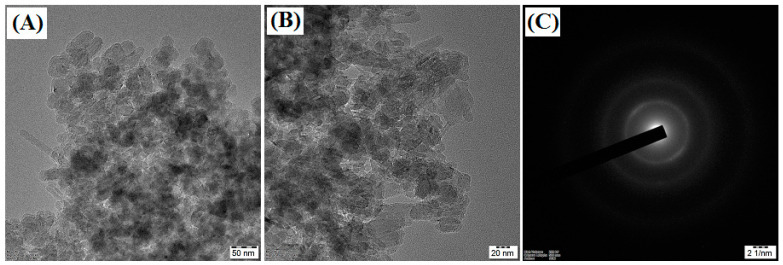
(**A**,**B**) HRTEM image of CNP/EDTAPEI sorbent at varying resolution illustrates the surface morphology of nanofibers; (**C**) SAED pattern of CNP/EDTAPEI sorbent.

The ATR-FTIR spectra of CNP/EDTAPEI sorbent (Figure 2) show the characteristic peaks at 3300, 1610, 1512, 1265 and 1031 cm^−1^, corresponding to structural –OH, –C=O,–NH, –CO and –CN stretching vibrations of surface functional groups [34,35]. The CNP/EDTAPEI after metal ions adsorption were characterized by FTIR and showed a minor shift with a decrease in peak intensity, as shown in Figure 2. Figure 3A–C shows the TEM image of the CNP/EDTAPEI at different resolutions. It was observed that the sorbent shows the particle structure after ligand incorporation into cellulose units. The random distribution of nanofibers in the TEM image indicates particle formation. The sorbent shows amorphous nature ascribed to the selected area electron diffraction (SAED) pattern obtained from the TEM image (Figure 3C). The multi-point BET surface area of CNP/EDTAPEI observed after N_2_ sorption-desorption was found to be 254.8 m^2^ g^−1^.

### 3.2. Optimization of Sample pH

**Figure 4:**
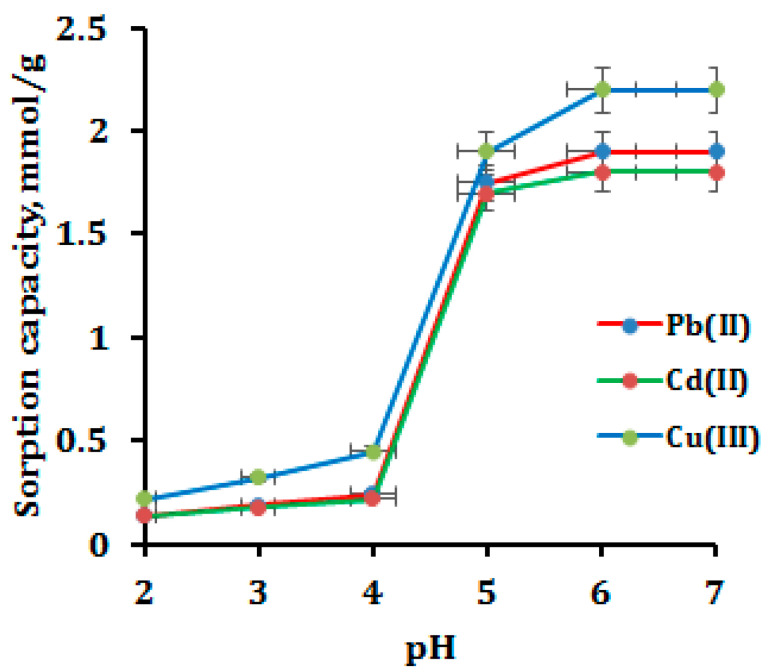
Effect of sample pH on the adsorption of metal ions.

It is well known that the pH of the sample solution affects the surface charge of the adsorbent. Herein, we studied the adsorption of Pb(II), Cd(II) and Cu(II) at pH 2–7; beyond pH 7.0, they would form a precipitate. The adsorption trend of metal ions as a function of sample pH is shown in Figure 4. It is observed that, at low sample pH (pH 2–4), the adsorbent surface becomes protonated and becomes positively charged, resulting in low adsorption of metal ions. This is due to the fact that the positively charged surface has a weak electrostatic interaction between the chelating sites and divalent metal ions [36]; however, when increasing the sample pH, the adsorbent surface becomes deprotonated, and the surface becomes negatively charged, thus easily forming metal chelates at high sample pH 5–7. Beyond pH 7, at a basic medium (pH 8–10), the metal ions become precipitated and form hydroxides. The functionalized CNP showed maximum adsorption at a pH range of 5−7 and much less adsorption at pH 2–4. The adsorption of Cu(II) onto CNP/EDTAPEI occurs via the chelation of nitrogen-containing functional groups [37], whereas Pb(II) and Cd(II) formed complexes onto CNP/EDTAPEI, probably at oxygen-rich active sites along with nitrogen [37]. This is also in accordance with hard, soft, acid and base theory [38,39]. Moreover, the prepared sorbent simultaneous adsorption of all three studied metal ions in a wider pH range due to the availability of various combinations of functional groups. In addition, to sample preparation, the CNP/EDTAPEI adsorbents can be used for the simultaneous removal of heavy metal ions at a common pH value of 5−6, thus making it useful in water filtration and purification.

### 3.3. Optimization of Contact Time

**Figure 5:**
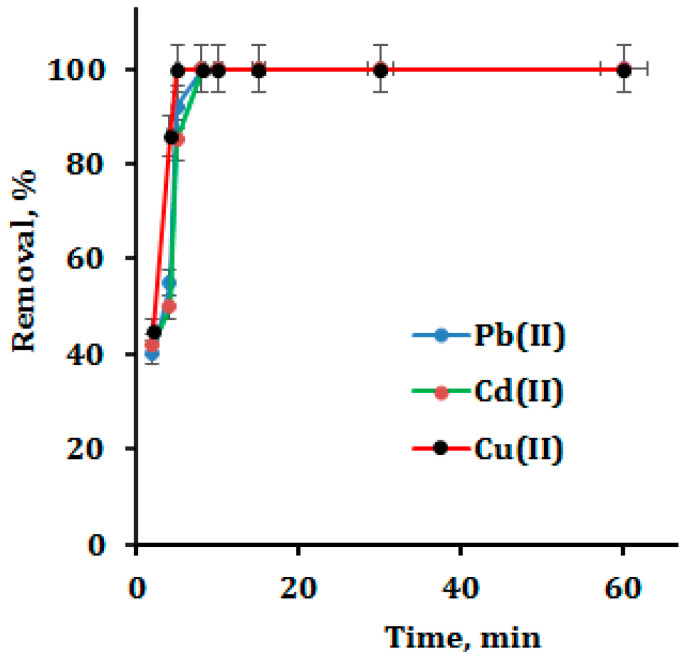
Effect of contact time on metal ion adsorption.

The sorbent–sorbate contact time was investigated and improved in order to obtain maximal adsorption. In an Erlenmeyer flask, 50 mg of CNP/EDTAPEI was agitated for 2 to 60 min at pH 6 with 50 mL of metal ions (10 mg L^−1^). ICP-OES was used to evaluate the metal ion that was left in the supernatant. The data collected are shown in Figure 5. It was discovered that Pb(II) and Cd(II) were entirely removed after 8 min of equilibration, while Cu(II) was eliminated after a shorter contact period of 5 min. This may be because the nitrogen functionality has a strong propensity to attach to Cu(II), then Pb(II) and Cd (II) [37,40,41]. Because of its high chelating efficiency and good accessibility to metal ions, the CNP/EDTAPEI exhibits quicker metal ion adsorption. This potential use in the preconcentration of trace metal ions from actual samples is suggested by its quicker adsorption rate.

### 3.4. Effect of Column Flow Rate

**Figure 6:**
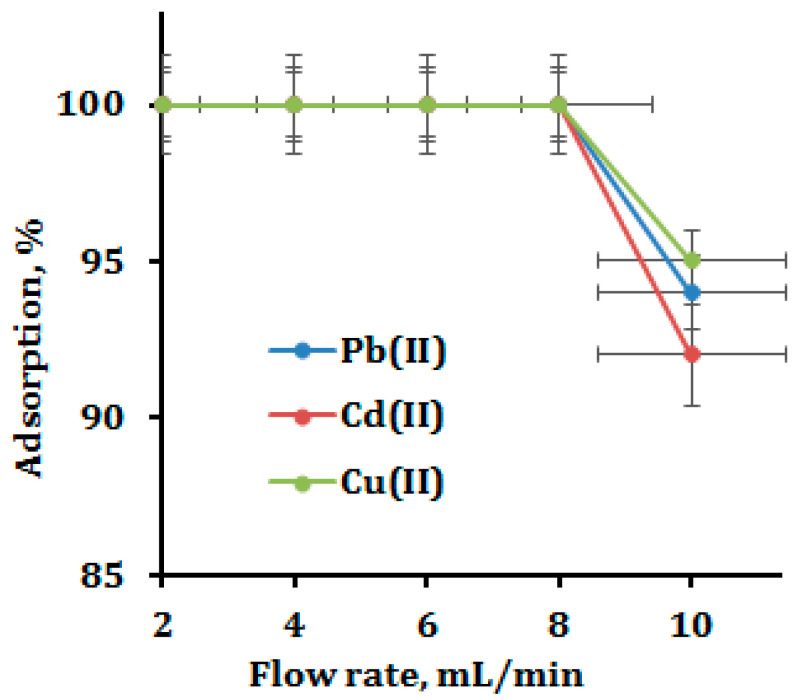
Effect of sample flow rate.

Sample flow rate optimization is equally significant to other parameters. The ideal sample flow enables full metal ion extraction from intricate matrices. Model solutions (vol. 100 mL; metal ion 10 µg; pH 6.0) were repeatedly cycled through the column at varied flows between 2- and 10-mL min^−1^ in order to analyze and improve the sample flow in the column. Figure 6 displays the plotted and acquired findings. Up to a flow rate of 8 mL min^−1^, which is among the highest reported cellulose-based column experiments described in the prior literature, the entire extraction of feed metal ions (Pb, Cd and Cu) was unaffected. Given the rapid flow rate and full extraction of metal ions, functionalized CNP must have a high affinity. The recovery of metal ions gradually declined by 5–10% above the 8 mL min^−1^ flow rate. Such decreased adsorption at greater sample flows may be caused by insufficient metal ion interaction with the functionalized CNP’s active sites. For the column adsorption tests, a sample flow rate of 8 mL min^−1^ for all the metal ions was chosen and used.

### 3.5. Type of Eluent

**Figure 7:**
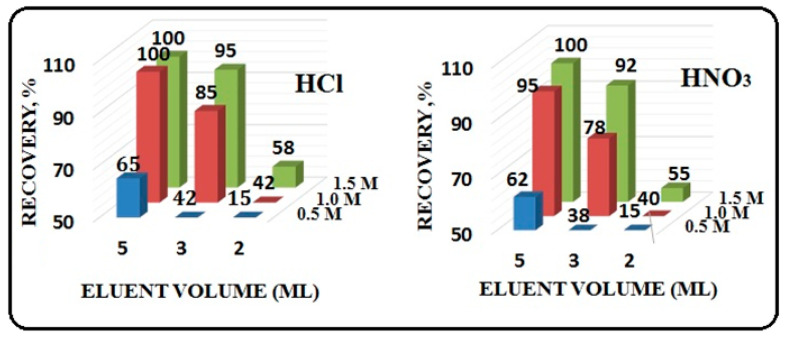
Optimization of eluent type, volume and concentration.

A crucial factor in accurately estimating the trace analyte ion concentrations in the solution is the quantitative recovery of the adsorbed analyte ions. A series of elution studies were conducted using functionalized CNP adsorbents to remove the analyte ions that had been retained. Figure 7 displays the outcomes for the elution of sorbed metal ions by various mineral acids, including nitric acid (HNO_3_) and hydrochloric acid (HCl), at various volumes (2–5 mL) and concentrations (0.5–1.5 M). We found that 5 mL of 1 M HCl was high enough to completely desorb (with a recovery rate of >99%) all analyte ions. In contrast, 85% of the sorbed metal ions are eluted by 3 mL of 1 M HCl. Then, 5 mL of hydrochloric acid was utilized as the eluting agent for additional research. Using the same column bed for many adsorption–elution tests, the reusability of functionalized CNP was examined. Up to the 60th cycle, the column was successfully employed, and after that, a progressive decline in sorption was seen. In particular, a 10–15% decline in all examined metal ion recoveries was seen in the cycles after 60. This decrease in recovery could be brought on by ligand leaching or degradation from the CNP sorbent. In conclusion, it was possible to extract and preconcentrate metal ions using functionalized CNP composites in many cycles.

### 3.6. Effect of Interferents 

Real wastewater samples frequently contain alkali and alkaline earth metals, which can interfere with the preconcentration of analytes. Cationic matrices typically compete with the analytes for the same adsorbent binding sites, reducing the target ion’s capacity to adsorb. In addition, spectral interference in the ICP-OES analysis is brought on by these coexisting ions with samples. The adsorption performance of the functionalized CNP must therefore be assessed under competitive conditions. The ratio of the interferents to the analyte was carefully examined for different co-existing alkali and alkaline earth metal ions. In order to perform this, 100 mL of sample solutions, each containing 10 µg of Cu(II), Pb(II) and Cd(II), as well as significant amounts of interfering ions, are kept at pH 6 and run through the functionalized CNP column at a rate of 8 mL per minute. Cu(II), Pb(II) and Cd(II) preconcentration showed no notable effects since the recovery for all the investigated ions was greater than 97% (Table 1).

### 3.7. Preconcentration Studies

In order to determine the lowest analyte concentration at which the quantitative recovery can be achieved, a bench of model solutions containing 1 g of metal ions (fixed amount) in differing volumes of 1000, 1200, 1400, 1500 and 1600 mL were percolated through the column under the ideal conditions. The adsorbed metal ions were eluted and then measured by ICP-OES after the column was washed with deionized water. The relevant information is explained in Table 2. Metal ions were quantitatively recovered up to a sample volume of 1400 mL (99.8%), but as the volume was increased to 1500 mL, the quantitative recovery fell to 90%. At 1500 mL of sample volume, the recovery of Cu(II) was reduced to 92%. Similar to Pb(II), Cd(II), analytes were recovered up to a sample volume of 1400 mL before being recovered at a rate of 87–90%. For Cu(II), Pb(II) and Cd(II), the highest preconcentration limit was 0.7 µg L^−1^, with a corresponding preconcentration factor of 280. With this preconcentration limit, the material was found suitable for application in the precise determination of analytes. Since more quickly reaching equilibrium between functionalized CNP and the metal ions in aqueous solutions is made possible by the hydrophilicity of CNP, this increases the enrichment efficiency.

### 3.8. Analytical Method Validation and Real Sample Analysis

By evaluating key factors such as linear range, relative standard deviation, detection limit (LOD) and accuracy of results, the presented approach has been validated. After preconcentrating the standard solutions (100 mL) for studied metals, the calibration curve was plotted using the least squares method, and it was discovered to be linear with the correlation coefficient (R^2^) equal and/or closer to 0.9999 (Table 3). After analyzing replicate samples of size, with 100 mL possessing 5 µg of each metal ion, the precision of the procedure was assessed. Replicate measurements’ coefficients of variation were discovered to be between 4 and 5%, demonstrating the method’s high degree of precision. According to International Union of Pure and Applied Chemistry [42], LOD is defined as 3 S/m of blank signal for 10 repeated evaluations of metal ions; this value was found as 0.40 µg L^−1^. By examining SRMs, the accuracy of the devised procedure was evaluated. The essential Student’s t value for N = 3 was found to be 4.303; however, the observed values were less than that. 

Table 4 provides an illustration of these outcomes. No systemic technique mistakes were discovered since, even in the presence of other concomitants, when matched to certified values, the average concentration values obtained using the specified method were statistically insignificant. Another crucial element for the suggested approach is reliability, which was investigated by adding a known quantity of analytes (5 µg) to genuine water and food samples. The percentage yields for the elevated concentrations were examined, and the maximum results had an relative standard deviation (RSD) of 5% (Table 5). By adjusting the solution’s optimal pH with an alteration of ±0.5 and flow rate to ±0.5 mL min^−1^, the robustness of the method was tested, and no appreciable changes in the recovery (>96%) for any of the analyte ions were discovered.

Enrichment and detection of trace amounts of Cu(II), Pb(II) and Cd(II) from industrial effluent, river water, groundwater and tap water samples were effectively accomplished using the established approach. The observed data are shown in Table 5. By adding known concentrations of analytes (5 µg) to the samples, the accuracy of the procedure was evaluated. The recoveries of the analyte ions were calculated by analyzing the recovered amount (spiked) from actual samples. The quantification of the ingested metal ions with a 95% confidence level was discovered. The examined metal ions’ average percentage recoveries ranged from 98.0 to 100.2%, with an RSD for the metal ions that were spiked being less than 5%. Moreover, comparative data of adsorption capacity, enrichment factor and limit of detection of this work with previous reports have been presented in Table 6.

## 4. Conclusions

A novel solid phase adsorbent made by covalently functionalizing cellulose nanofibers was used in a column technique. The effectiveness of preconcentration and metal ion adsorption are both greatly enhanced by increasing the functional group density. The method shows efficient adsorption of analytes at an optimum pH value of 6.0 and at a flow rate 8 mL min^−1^ with a preconcentration limit of 0.7 ppb. The suggested method could be utilized to preconcentrates trace analytes from real water samples at neutral pH levels. According to EPA and WHO criteria, the detection limit obtained for the examined metal ions (0.4 µg L^−1^) was far lower than the maximum contamination level that can be found in drinking water (5–10 µg L^−1^). Therefore, it is possible to use the established approach for routine analysis of trace metal ions in drinking water samples. The evaluations of the accepted reference materials accurately and precisely verified the method’s dependability. In actual water samples, when speciation and preconcentration are complicated by the presence of other coexisting ions, the approach has been shown to work well.

## Figures and Tables

**Table 1:** Interference studies.

Co-Existing Ions	Salt Added	Amount Added (×10^3^ µg L^−1^)	Recovery % (RSD)		
			Pb(II)	Cd(II)	Cu(II)
Na^+^	NaCl	580	99.4 (4.15)	97.9 (4.75)	99.9 (3.52)
K^+^	KCl	540	99.5 (4.65)	98.7 (4.90)	99.6 (4.15)
Ca^2+^	CaCl_2_	75	99.6 (4.28)	98.5 (3.15)	99.4 (3.83)
Mg^2+^	MgCl_2_	120	98.9 (3.34)	98.5 (4.16)	99.3 (3.94)
Cl^−^	NaCl	980	99.3 (4.16)	98.6 (4.12)	98.6 (3.56)
Br^−^	NaBr	820	98.7 (3.14)	98.8 (4.12)	99.6 (2.44)
CO_3_^2−^	Na_2_CO_3_	340	97.9 (4.56)	97.5 (4.15)	98.3 (3.39)
SO_4_^2−^	Na_2_SO_4_	220	97.6 (4.15)	98.5 (3.32)	97.5 (2.34)
NO_3_^−^	NaNO_3_	300	99.5 (2.05)	97.3 (3.65)	99.5 (4.05)
CH_3_COO^−^	CH_3_COONa	320	98.9 (2.96)	98.5 (3.05)	97.2 (3.96)
C_6_H_5_O_7_^3−^	Na_3_C_6_H_5_O_7_	260	98.8 (4.15)	98.5 (3.42)	98.5 (3.34)
Humic acid	-	20	97.2 (3.54)	97.8 (3.24)	97.6 (3.64)
Fulvic acid	-	20	98.5 (3.05)	97.6 (4.12)	97.5 (4.05)
Fe(II)	FeCl_2_	1.2	97.7 (4.21)	97.0 (3.98)	98.6 (3.88)
Fe(III)	FeCl_3_	1.5	99.0 (2.88)	98.7 (4.76)	98.2 (3.56)
As(III)	AsCl_3_	1.5	99.4 (3.76)	99.7 (4.03)	98.9 (3.84)
As(V)	AsOCl	2.0	99.0 (4.42)	98.7 (3.49)	98.7 (4.72)
Hg(II)	HgCl_2_	1.8	97.8 (4.33)	98.5 (2.98)	98.0 (4.06)

**Table 2:** Preconcentration studies data for CNP/EDTAPEI (column parameters: M^n+^ = 1.0 µg, pH 6, flow rate 8 mL min^−1^, eluent 5 mL of 1M HCl, sorbent amount 100 mg).

Sample Volume (mL)	Analyte Concentration (µg L^−1^)	Preconcentration Limit			Preconcentration Factor		
		Cu(II)	Pb(II)	Cd(II)	Cu(II)	Pb(II)	Cd(II)
1000	1.0	1.0	1.0	1.0	200	200	200
1200	0.83	0.83	0.83	0.83	240	240	240
1400	0.71	0.71	0.71	0.71	280	280	280
1500	0.66	-	-	-	-	-	-

**Table 3:** Regression equation for the calibration curve (concentration range of 1–5000 μg L^−1^).

Metal Ion	Regression Equation	R^2^
Cu(II)	A = 19.4708 X_Cu_ + 3.0125	0.9999
Pb(II)	A = 109.6104 X_Pb_ + 4.1675	0.9997
Cd(II)	A = 9.8504 X_Cd_ + 4.1525	0.9996

**Table 4:** Analytical method validation.

Samples	Certified Values (µg g^−1^)	Values Found by Proposed Method (µg g^−1^) ^a^	Value of *t*-Test ^b^
NIES 10C	Cd: 1.82; Cu: 4.1	Cd: 1.84 ± 0.008; Cu: 4.2 ± 0.041	1.150; 1.242
SRM 1572b	Pb: 13.3; Cu: 16.5	Pb: 12.8 ± 0.082; Cu: 16.2 ± 0.057	1.126; 1.312

^a^ Mean value ± standard deviation, N = 3; ^b^ at 95% confidence level.

**Table 5:** Determination of metal ions in environmental water samples using ICP-OES after column preconcentration (column parameters: sample flow rate 8 mL min^−1^, eluent volume 5 mL (HCl) and eluent flow rate 1 mL min^−1^).

Samples	Amount Added (µg)	Metal Ion Found (µg L^−1^) ± Standard Deviation ^a^ (% Recovery of Added Amount; RSD)		
		Cu(II)	Pb(II)	Cd(II)
Industrial effluent	0	18.5 ± 0.52	7.30 ± 0.85	3.57 ± 0.56
	5	23.4 ± 0.63 (98; 3.35)	12.30 ± 0.61 (100; 4.19)	8.56 ± 0.89 (99.8; 3.87)
Groundwater	0	10.5 ± 0.41	2.8 ± 0.02	2.4 ± 0.09
	5	15.4 ± 0.22 (98; 1.58)	7.8 ± 0.37 (100; 1.68)	7.42 ± 0.31 (100.4; 2.14)
Tap water	0	3.7 ± 0.27	2.2 ± 0.08	3.1 ± 0.31
	5	8.7 ± 0.16 (100; 2.03)	7.24 ± 0.12 (100.8; 1.34)	8.12 ± 0.40 (100.4; 1.56)
River water	0	nd ^b^	1.1 ± 0.02	nd ^b^
	5	4.99 ± 0.15 (99.8; 2.87)	6.08 ± 0.16 (99.6; 4.05)	5.06 ± 0.38 (101.2; 2.53)

^a^ N = 3; nd ^b^ not detected.

**Table 6:** Adsorption of metal ions by different cellulose-based adsorbents.

Adsorbent	Adsorption Capacity (mg g^−1^)	Preconcentration Factor	Detection Limit (µg L^−1^)	Ref.
BC-PEI	Pb: 148; Cu: 141	-	-	[43]
APBC	Pb: 103; Cd: 76; Cu: 108	Pb: 540; Cd: 540; Cu: 580	Pb: 0.05; Cd: 0.04; Cu: 0.03	[44]
BC/PVA/GO/APT	Pb: 218; Cu: 151	Pb: 150; Cu: 150	-	[45]
Cell-EDTA and Cell-CM	Pb: 41.2, 33.2; Cd: 63.4, 23.0	-	-	[46]
CNP-EDTAPEI	Pb: 393; Cd: 202; Cu: 139	Pb, Cd, Cu: 280	Pb, Cd, Cu: 0.4	This work

## Data Availability

Not Applicable.
